# Exercise and nutrition in type 1 diabetes: Insights from the FinnDiane cohort

**DOI:** 10.3389/fendo.2022.1064185

**Published:** 2022-12-22

**Authors:** Drazenka Pongrac Barlovic, Valma Harjutsalo, Per-Henrik Groop

**Affiliations:** ^1^ University Medical Center Ljubljana, Department of Endocrinology, Diabetes and Metabolic Diseases, Ljubljana, Slovenia; ^2^ Faculty of Medicine, University of Ljubljana, Ljubljana, Slovenia; ^3^ Folkhälsan Institute of Genetics, Folkhälsan Research Center, Helsinki, Finland; ^4^ Department of Nephrology, Helsinki University Central Hospital, University of Helsinki, Helsinki, Finland; ^5^ Research Program for Clinical and Molecular Metabolism, University of Helsinki, Faculty of Medicine, Helsinki, Finland; ^6^ Department of Diabetes, Central Clinical School, Monash University, Melbourne, VIC, Australia

**Keywords:** type 1 diabetes, physical activity, diet, depression, complications, review

## Abstract

Type 1 diabetes is a challenging disease, characterized by dynamic changes in the insulin need during life periods, seasons of the year, but also by everyday situations. In particular, changes in insulin need are evident before, during and after exercise and having meals. In the midst of different life demands, it can be very burdensome to achieve tight glycemic control to prevent late diabetes complications, and at the same time, to avoid hypoglycemia. Consequently, many individuals with type 1 diabetes are faced with diabetes distress, decreasing profoundly their quality of life. Today, the nationwide Finnish Diabetic Nephropathy (FinnDiane) Study, launched in 1997, has gathered data from more than 8,000 well-characterized individuals with type 1 diabetes, recruited from 93 centers all over Finland and has established its position as the world’s leading project on studying complications in individuals with type 1 diabetes. Studying risk factors and mechanisms of diabetes complications is inconceivable without trying to understand the effects of exercise and nutrition on glycemic control and the development of diabetes complications. Therefore, in this paper we provide findings regarding food and exercise, accumulated during the 25 years of studying lives of Finnish people with type 1 diabetes.

## 1 Introduction

In no other disease, day-to-day self-management is as important as it is in type 1 diabetes to prevent acute and chronic complications. Besides the 21st century modern technology, including continuous glucose monitoring (CGM) systems and algorithm-controlled insulin delivery systems based on real-time CGM, active self-management is suboptimal. Glucose control and diabetes outcomes are still dependent on food choices, engagement in physical activity, but also on knowledge and the ability for empowering people to actively participate in decision making regarding their self-care (1, 2). In line with this, there is an array of individual factors, such as motivation, self-efficacy, coping skills, locus of control, sense of coherence (1), psychological characteristics, but also environmental factors, e.g., social support, factors related to provider of care, that influence the management and outcomes of type 1 diabetes (1, 2).

In the present paper we provide data on this topic from research conducted within the FinnDiane cohort during the 25 years since the study was launched in 1997. FinnDiane is one of the world’s largest cohorts of individuals with type 1 diabetes from the country with the highest incidence of type 1 diabetes. Our research has focused on studying the effects of physical activity, diet and psychosocial variables on diabetes complications and mortality.

## 2 Physical activity - associations with glycaemic control, chronic complications and mortality

At the cross-sectional level, we have shown that low level of leisure time physical activity (LTPA), e.g., less than 2 hours of walking per week, was associated with glucose concentration outside the target range in women with glycated hemoglobin (HbA_1c_) higher than 8,5% after adjustment for numerous confounding factors. In men, on the other hand, increased LTPA level was associated with improved estimated insulin sensitivity (3). Our data also suggest that higher intensities of physical activity confer no additional benefit on the HbA_1c_ level. Therefore, our hypothesis was, that LTPA does not follow a general dose-response curve regarding glycemic control in type 1 diabetes. Rather, avoidance of sedentary behavior is crucial for having glycemic benefits.

Moreover, we have shown that individuals with different chronic complications differed in their LTPA patterns (4). Low intensity of LTPA was more common in individuals with diabetic nephropathy, retinopathy and cardiovascular disease (CVD). This was expected, since people with various complications, like severe retinopathy, foot ulcers and angina pectoris are advised to limit their physical activity or lower its intensity. However, a decrease in the intensity of LTPA was seen already at the level of microalbuminuria (moderate albuminuria), whereby individuals with microalbuminuria more frequently reported low-intensity LTPA than those with normal albumin excretion rate (4). Microalbuminuria in type 1 diabetes is unlikely to cause exercise intolerance, because persons with microalbuminuria usually have normal kidney function, and do not have anemia, which might impair oxygen delivery to the muscles during exercise. Therefore, our findings suggest that low intensity of LTPA precedes the development of microalbuminuria and potentially also the development of other chronic complications.

**Figure 1:**
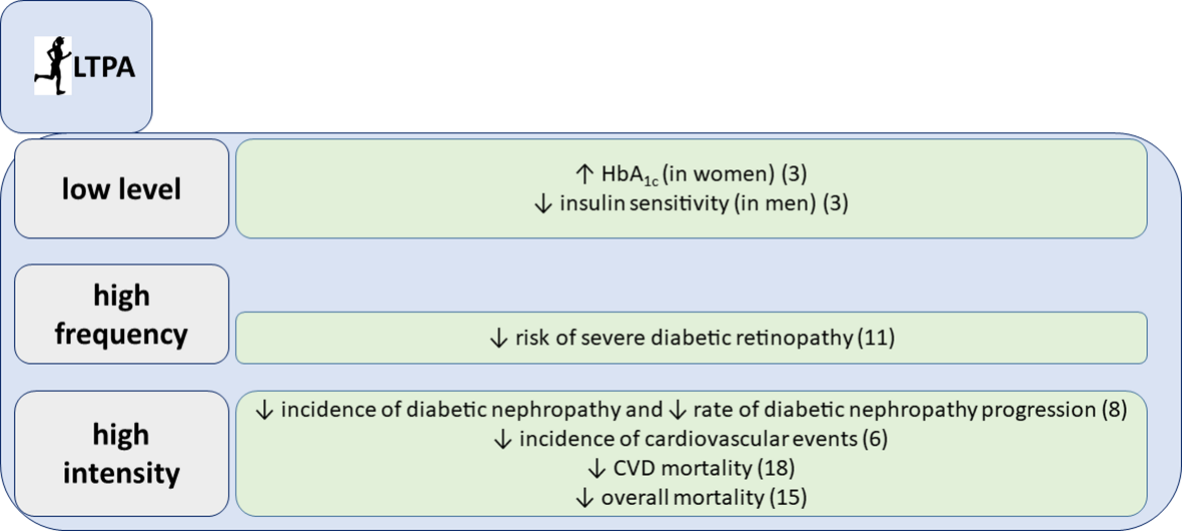
Associations of leisure time physical activity (LTPA) with glycemic parameters and chronic complications, summarized findings from the FinnDiane study.

Although beneficial effects of physical activity on mortality and cardiovascular events are well recognized in the general population, this may not directly be translated to the type 1 diabetes population. Indeed, prospective data from the EURODIAB study could not confirm any positive association with CVD events or mortality (5). In our prospective studies, however, we have consistently shown that physical activity is associated with reduced risk of the development of chronic complications and premature mortality (**Table 1**; **Figure 1**).

**Table 1:** Summary of FinnDiane data showing the effect of different components of leisure-time physical activity (LTPA) and development of chronic complications or mortality (6–11).

LTPA	Cardiovascular disease	Diabetic nephropathy	Diabetic retinopathy	MORTALITY	
				ALL	CVD
**TOTAL**	**+**	**-**	**-**	**+**	**-**
**Intensity**	**+**	**+**	**-**	**+**	**+**
**Frequency**	**+**	**+**	**+**	**+**	**+**
**Duration**	**+**	**-**	**-**	**+**	**-**

LTPA was assessed by a self-report questionnaire, whereby exercise intensity was considered as low (no self-reported subjective shortness of breath and no sweating), moderate (a moderate degree of self-reported subjective shortness of breath and sweating), high (a high degree of subjective shortness of breath and sweating). Exercise frequency was considered as low (fewer than one session per week), moderate (one to two sessions per week), high (more than two sessions per week). Exercise duration was defined according to duration of a single session of physical activity as low (≤30 min/session), moderate (31–60 min/session) and high (>60 min/session).

We followed for 10 years 2,180 individuals with type 1 diabetes and collected comprehensive data on frequency, intensity and duration of LTPA. We could show that LTPA, especially the high frequency and high intensity, reduced the risk of CVD events, even after adjustment for classic risk factors (6). It may be somehow unexpected, since both higher frequency and intensity of LTPA may also increase the risk of hypoglycemia and glucose variability. However, our results are in line with recent findings from a large observational study in the general population, underlying that the greatest mortality benefit can be seen at the level of moderate and vigorous activity that is two-to-four times higher than the amount currently recommended by the national guidelines (7).

Since CVD shares common risk factors with diabetic kidney disease and since they usually develop in parallel, it was not surprising that we found that physical activity, particularly its intensity, lowers the risk of progression of diabetic nephropathy in type 1 diabetes (8). Even more importantly, we have demonstrated that intensive physical activity prevents the initiation of diabetic nephropathy (8). These findings support the current recommendations for chronic kidney disease management and suggest that exercise should always be a part of the treatment regimen (12, 13).

Likewise, we have shown that frequent physical activity during 10 years of follow-up reduces the risk of severe diabetic retinopathy, even after adjustments for multiple confounders in individuals with an average type 1 diabetes duration of almost 20 years (9). In our analysis, however, higher intensity LTPA was associated with an increased cumulative incidence of severe diabetic retinopathy. Overall, there is a lack of mechanistic studies about the effects of intensive physical activity on diabetic retinopathy, and it is of note that the characterization of diabetic retinopathy status was not particularly meticulous in our study. Nevertheless, it has been shown that autoregulation is impaired in people with diabetes and especially in cases with glucose concentration outside the target range and microvascular damage (14). In such individuals, intensive exercise and the concomitant increase in blood pressure may lead to retinal damage due to impaired autoregulation. Our observations are in line with the current American Diabetes Association clinical practice recommendations, stating that vigorous intensity physical activity or resistance exercise are contraindicated in case of proliferative diabetic retinopathy, because of the risk of triggering vitreous hemorrhage or retinal detachment (15). Nevertheless, further studies are needed to clarify whether regular physical activity and potential hemodynamic adaptations could lead to improved vascular reactivity (16).

We have also analyzed the association between various aspects of LTPA and mortality in individuals with an average type 1 diabetes duration of 23.3 ± 12.8 years during a mean follow-up time of 11.4 ± 3.5 years. LTPA and all its components were associated with lower all-cause mortality, even after adjustment for the potential confounders sex, diabetic nephropathy, duration of diabetes, age at onset of diabetes, systolic blood pressure, triglycerides, BMI, and HbA1c (10). Moreover, we have shown that also in individuals with diabetic nephropathy, which is one of the main determinants of premature mortality in type 1 diabetes (11), total LTPA as well as all its components were associated with decreased all-cause mortality, independently of a series of confounders (10). The finding is not unexpected given that we have previously shown that exercise, and in particular intensive exercise, is associated with reduced risk of progression of diabetic nephropathy (8). Individuals with chronic kidney disease are expected to be physically more inactive and to have a lower level of fitness and muscle function (17), but exercise appears to be safe and to have significant health benefits also in these individuals.

Similarly, exercise intensity was associated with reduced cardiovascular mortality after adjustment for the confounders in our cohort (10). Given that cardiovascular disease is the most common cause of death among individuals with type 1 diabetes (18), it is not surprising that exercise intensity is also associated with lower risk of cardiovascular mortality. The reason why only intensity remains significant after adjustment for various confounders is not known. One possibility is that confounders like blood pressure, glycemic control and lipid concentration are themselves affected by exercise and thus their association is lost after statistical adjustment. Another hypothesis is that exercise intensity is the most important component of LTPA because of its impact on heart rate and autonomic nervous function. We have previously shown that individuals with type 1 diabetes display reduced baroreflex sensitivity (19), which is clinically detectable as a higher pulse rate and, thus, potentially reduced exercise tolerance and fitness. Intriguingly, we have shown that already with a simple intervention, like slow deep breathing, improvements in baroreflex sensitivity can be induced in most individuals with type 1 diabetes, irrespective of the duration of diabetes (19, 20).

One of the major barriers to physical activity is fear of hypoglycemia and self-management skills (21). When we assessed fear of hypoglycemia in our FinnDiane cohort, we saw that it was more common in women (in 62% of women and 43% of men fear of hypoglycemia was observed). In men, it was associated with higher mean serial HbA1c level, higher carbohydrate intake and lower high-fat meal intake, whereas in women, it was associated with higher energy intake. Additionally, both men and women with fear of hypoglycemia more frequently monitored their blood glucose concentrations (22). Of note, higher sense of coherence, defined as greater self-confidence that an individual has sufficient resources to meet the demands of life, was associated with higher levels of physical activity in men and predicted healthier food choices in women in our cohort (1).

## 3 Nutrition - meeting diet recommendations, glycemic control and chronic complications

**Figure 2:**
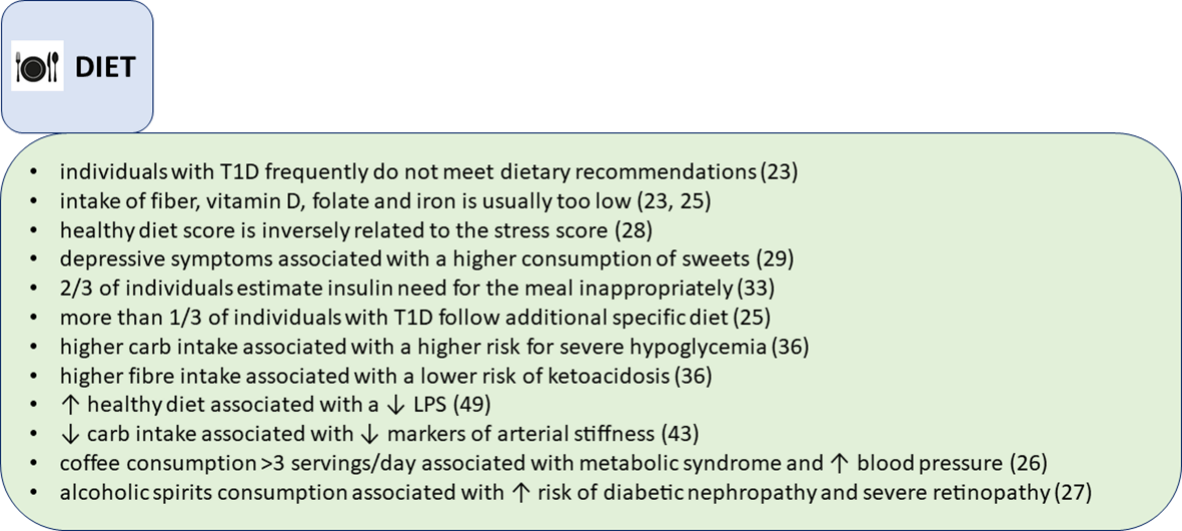
Findings on nutritional parameters, summarized from the FinnDiane study.

It is hard to find a disease, where diet plays a more central role in the disease control and the disease outcomes than type 1 diabetes. However, we have shown that Finnish adults with longstanding type 1 diabetes (average diabetes duration 33 years), do not, for many nutrients, meet the dietary recommendations (15) according to their self-reported dietary intake (23). In our study, more than 70% of individuals with type 1 diabetes exceeded the recommendations for saturated fat and salt intake (**Figure 2**). Fiber intake was below the recommendations in almost all individuals, and one-fourth exceeded the recommendations for sucrose intake. Moreover, a large proportion reported diets with low vitamin D, folate, and iron intake (23). When compared to the general population in Finland, our study population had similar diet composition, except for lower carbohydrate intake (24). Interestingly, individuals who considered themselves compliant regarding macronutrient intake in our analysis, achieved the guideline recommendations only modestly (e.g., in 88% for protein, 77% for sucrose, 6% in fiber intake). This might suggest that individuals with diabetes need more education on the desirable eating patterns, but even more likely, it might reflect the fact that it is not easy to change dietary practices from the established ones in a certain environment. Since scrutinizing dietary choices may be overwhelming in everyday life, maybe a more widespread use of certain tools that help monitoring daily macronutrient intake (i.e., mobile food applications), could help in approaching healthier dietary patterns.

Interestingly, we observed that a relatively high proportion of our FinnDiane cohort followed some sort of additional diet, e.g., lactose-free, vegetarian, gluten-free, protein restriction or others (25). Those were mainly women with longer diabetes duration and more diabetes complications. Adherence to gluten-free diet was associated with the lowest frequency of meeting the diet recommendations, while vegetarians and those with protein intake restriction due to chronic kidney disease achieved the highest scores in meeting the diet recommendations. The recommendations for folate, iron, vitamin D and fiber intake were frequently unmet in all special diet groups (25).

We also analyzed the associations of popular drinks, like coffee and alcohol consumption in type 1 diabetes. Contrary to observations in the general population where habitual coffee consumption is inversely associated with the metabolic syndrome, we found that consuming 3 or more cups of coffee per day is positively associated with the metabolic syndrome in type 1 diabetes (26). Moreover, any level of coffee consumption was associated with an increased blood pressure (26). Also, in another cross-sectional analysis, we examined how alcohol consumption affects people with type 1 diabetes. In a study including more than 3000 FinnDiane participants, consumption of alcoholic spirits was associated with a higher risk of diabetic nephropathy and severe retinopathy compared with wine drinkers (27).

In our studies we have observed that psychological factors frequently affect diet choices. For example, stress score was negatively associated with a healthy diet score, independently of the body mass index (BMI) (28). In individuals with normal BMI, higher stress score was associated with worse glycemic control. However, in individuals with overweight or obesity, higher glucose concentrations were present regardless of the level of perceived stress (28). Also, depressive symptoms were associated with an increase in the “sweet” diet pattern, characterized by an increased consumption of sweet pastry, sweets, chocolate and ice cream. On the other hand, those with the lowest scores regarding depressive symptoms preferred proteins over fats and carbohydrates (29). We have also found that higher depression scores are reflected in a higher prevalence of the metabolic syndrome (30), more inactive lifestyle, lower frequency of weekly LTPA sessions, lower intensity of the physical activity, and shorter duration of the exercise sessions (31). Of concern, women who purchased antidepressant agents were found to have higher mortality rates in the FinnDiane study, with the most frequent underlying cause of death being chronic diabetes complications (32). In those who did not purchase antidepressant agents, cardiovascular disease was the most common underlying cause of death (32).

**Figure 3:**
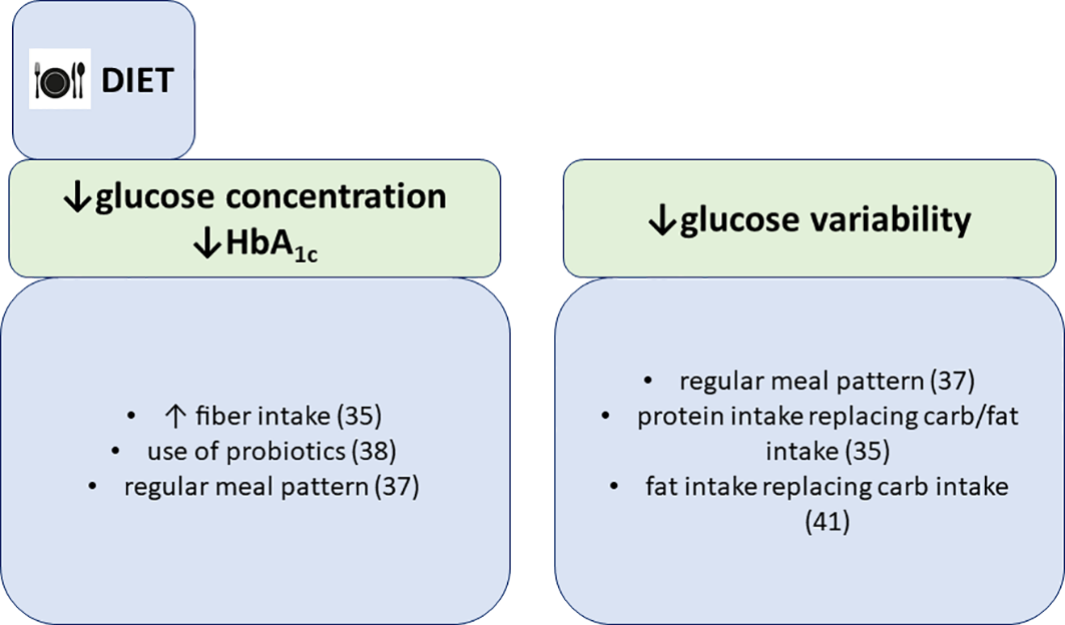
Associations of dietary patterns with glycemic parameters, summarized findings from the FinnDiane study.

What is more conspicuous is that food choices have a direct effect on the measured glucose concentrations in type 1 diabetes (**Figure 3**). We have reported that almost two thirds of persons with longstanding diabetes, included in our study, estimated their prandial insulin need inappropriately, resulting in hypoglycemia or hyperglycemia (33). Our findings support the hypothesis that matching prandial insulin dose with macronutrient content of the meal and planned physical activity is not easy, even after long duration of type 1 diabetes. This fact is also widely acknowledged by the professional community, and emphasis is focused on developing tools that could make those decisions easier. For instance, mobile applications that help to estimate carbohydrate content in meals are available. Moreover, new insulin pumps with hybrid closed loop system will further improve glycemic outcomes through automatic meal bolus adjustment based on glucose trend (34).

We have also demonstrated that reported dietary fiber intake is associated with lower mean self-monitored blood glucose concentration in our cohort of 1000 individuals (35). Furthermore, glycemic variability was lower if fat replaced carbohydrates and when protein substituted other macronutrients (35). Our results suggest that increasing fiber intake and exchanging fats and carbohydrates with protein could lead to better glucose control in type 1 diabetes. Our results were in line with another study of diet composition in individuals hospitalized for ketoacidosis, where those with high fiber intake were less likely to be hospitalized for ketoacidosis (36). Interestingly, persons with a higher median percentage of energy intake, derived from carbohydrates were more likely hospitalized for severe hypoglycemia (45,5% vs. 43,6% in those with hospitalization for severe hypoglycemia vs. those without, respectively). Unfortunately, only the amount of macronutrients was derived from the dietary assessments, by using AivoDiet software (version 2.0.2.3, AIVO, Turku, Finland). Thus we could not make additional conclusions about whether increased consumption of a specific type of carbohydrate was associated with the increased risk for severe hypoglycemia. Higher carbohydrate intake could well be the consequence of hypoglycemia, however, higher carbohydrate intake is very much dependent upon the right prandial insulin dose and as we have shown previously (33), people with type 1 diabetes have difficulties in determining the right bolus amount, thereby increasing the odds for hypoglycemia. In addition, we have also shown that a regular meal pattern, including breakfast consumption, is associated with lower HbA_1c_ and lower mean blood glucose concentration, however, with greater glucose variability (37).

In our studies we also investigated the association of microbiota and probiotics with glycemic control. We found that self-reported use of probiotics was associated with lower HbA_1c_ in normal weight individuals with type 1 diabetes (38). Although similar studies confirm the association of gut microbiome composition with glycemic control and diabetes complications (39), our findings may rather be a mere reflection of better self-care in individuals with probiotic consumption than a direct beneficial effect of probiotics on glucose metabolism, although several studies identified four key mechanisms linking the gut microbiota with host glycemic regulation. These mechanisms include increase of incretin secretion, bacterial production of short-chain fatty acids, bacterial metabolism of bile acids and modulation of lipopolysaccharide (LPS)-mediated inflammation and induction of white adipose tissue browning (40).

Diet, however, does not affect only glucose control. Dietary patterns with higher carbohydrate-to-fat ratio were associated with higher blood pressure, lower HDL-cholesterol concentration and in men, with lower waist-to-hip ratio in our study (41). In addition, low-carbohydrate diet, recently gaining popularity among individuals with type 1 diabetes, was associated with lower glucose variability, lower BMI and lower diastolic blood pressure, yet with higher cholesterol concentrations (41). Moreover, contrary to the traditional association of high fat consumption with poor vascular health (42), our results suggest that increasing fat intake over carbohydrates is associated with lower indirect markers of arterial stiffness, e.g., aortic mean arterial pressure, aortic pulse pressure and aortic augmentation index (43). Also, increasing protein intake over carbohydrates was associated with lower aortic mean arterial pressure and pulse wave velocity (43). The precise mechanism through which carbohydrates could contribute to arterial stiffness is unknown. Abundant carbohydrate intake is related to increased serum triglyceride concentrations and reduced concentrations of HDL-cholesterol (44). Nevertheless, our studies could not give an answer to the question whether the origin and the quality of fats and protein, replacing carbohydrates, play a role in the effect on the cardiovascular risk factors and mortality (45). We are aware of the fact that in these studies with diet recall and diet questionnaires, although validated, only memories of perceptions of dietary intake are captured, which may not be the snapshot of the actual caloric and nutrient consumption. Besides, identical diets consumed by different individuals can result in divergent metabolic and health effects, therefore without a detailed phenotyping, metabolic fate of consumed food and beverages remains largely unknown (46).

We have also reported that closer adherence to dietary recommendations in a group of male FinnDiane participants, was associated with lower high-sensitivity C-reactive protein (hs-CRP) (47), suggesting that dietary approaches might also be effective in direct reduction of low-grade systemic inflammation, thereby reducing the risk of late complications (48). The sex difference observed in the effect of diet score and inflammation is not completely understood, however, it could be a consequence of overall better diet in women, reaching higher diet scores, thereby masking the association between diet score and hs-CRP observed in men. Similarly, we found that individuals with healthier diet patterns, i.e., consumption of higher amounts of fish, vegetables, fruits and berries, have lower serum LPS activity (49), confirming data from the general population (50). LPS-induced secretion of proinflammatory cytokines can lead to endothelial dysfunction, atherosclerotic plaque formation, plaque rupture and thrombogenesis (51).

At a mechanistic level, we found that high-fat meals, with fats representing 44% to 58% of total energy intake, increased the serum levels of various pro-inflammatory cytokines (IL-6, TNF-alpha, IL-1beta, IFN-alpha, IFN-gamma, IL-10, IL-12, MIP-1beta) in individuals with type 1 diabetes as well as in controls, without concomitantly increasing the serum LPS activity (51, 52). Although most of the fat represented saturated fats, with less than 5% of energy intake from polyunsaturated fat, there was no postprandial change in serum cytokines production found, as well as no activation of circulating innate immune cells (monocytes or myeloid dendritic cells) detected (53). However, at fasting, myeloid dendritic cells in individuals with diabetes, exhibited higher LPS-induced IL-6 and IL-1 beta production than controls. We also found that after a high-fat meal, individuals with type 1 diabetes were unable to decrease the augmentation index and had a decreased activity of an antioxidant enzyme, paraoxonase (PON-1) (52). These data suggest that even though dietary fats have been shown to be able to promote translocation of bacterial endotoxins from the gut into the circulation, by modulating the inflammatory response the postprandial rise in cytokine level after high-fat meals is not immune-mediated, but rather regulated at the tissue level in type 1 diabetes.

Recently, we have also examined dietary patterns specifically across different kidney function strata. We have shown that already early in diabetic kidney disease, with estimated glomerular filtration rate (eGFR) only mildly reduced (60-89 ml/min/1.73m^2^), differences in eating patterns exist in type 1 diabetes (54). The general conclusion from our analysis was, that there is an overall trend towards healthier food choices upon advancing kidney disease, limiting salt intake and sweet food items (54). Importantly, caloric intake was significantly reduced already with mildly reduced glomerular filtration rate and although the average BMI was not in the range of obesity (≤27 kg/m^2^, in all eGFR groups). This may be related to following dietary recommendations to a greater extent and limiting the intake of unnecessary calories. Despite reduced caloric intake, BMI was not significantly lower, until the eGFR fell below 30 ml/min/1.73m^2^, possibly because healthier eating habit was accompanied by increased level of exercise, with concomitant increase in muscle mass and maintenance of BMI. However, with further deterioration in kidney function, BMI started to decline, suggesting that catabolic processes start to supervene. Nonetheless, detailed body composition studies are needed to confirm these hypotheses.

## 4 Relevance of these findings and concluding remarks

In this paper we have gathered the main insights from the vast research opus of the FinnDiane study analyzing how exercise and food patterns are associated with glycemic control, development of chronic complications and mortality. Phenotypically very well characterized participants included in one of the largest cohorts of individuals with type 1 diabetes with and without kidney disease, provide a useful platform for high quality observational research, especially in a prospective setting.

These data underline the importance of avoiding sedentary behavior for having glycemic benefits and emphasize the importance of physical activity, in particular high frequency and high intensity, for the reduction of risk of onset and progression of diabetic nephropathy, risk of CVD, CVD mortality and all-cause mortality. Our research has repeatedly shown that even in longstanding type 1 diabetes, healthy food choices and accurate prandial insulin dosing remain a challenge. Besides, our data suggest that increase in fiber intake and exchanging fats and carbohydrates with protein, could lead to better glucose control and decrease in arterial stiffness. Anyway, our research also suggests, that by using technology with its tools and by exploring psychosocial aspects, like increase in sense of coherence, to a greater extent, improvements in nutrition, physical activity engagement and diabetes outcomes could be observed.

## Author contributions

DP participated in the study conception, data interpretation and wrote the manuscript. VH participated in the data interpretation and critical revision of the manuscript. P-HG is the principal investigator of the FinnDiane study, participated in the study conception, data interpretation and critical revision of the manuscript for important intellectual content. All authors contributed to the article and approved the submitted version.

## Supplementary Material

The Supplementary Material for this article with a list of FinnDiane centers, participating in FinnDiane studies, can be found online at: https://www.frontiersin.org/articles/10.3389/fendo.2022.1064185/full#supplementary-material
